# Centralising and optimising decentralised stroke care systems: a simulation study on short-term costs and effects

**DOI:** 10.1186/s12874-016-0275-3

**Published:** 2017-01-10

**Authors:** Maarten M. H. Lahr, Durk-Jouke van der Zee, Gert-Jan Luijckx, Patrick C. A. J. Vroomen, Erik Buskens

**Affiliations:** 1 Health Technology Assessment, Department of Epidemiology, University of Groningen, University Medical Centre Groningen, Hanzeplein 1, P.O. Box 30001, 9700 RB Groningen, The Netherlands; 2 Department of Operations, Faculty of Economics & Business, University of Groningen, Groningen, The Netherlands; 3 Department of Neurology, University of Groningen, University Medical Centre Groningen, Groningen, The Netherlands

**Keywords:** Stroke, Simulation models, Organisational model, Costs, Thrombolysis

## Abstract

**Background:**

Centralisation of thrombolysis may offer substantial benefits. The aim of this study was to assess short term costs and effects of centralisation of thrombolysis and optimised care in a decentralised system.

**Methods:**

Using simulation modelling, three scenarios to improve decentralised settings in the North of Netherlands were compared from the perspective of the policy maker and compared to current decentralised care: (1) improving stroke care at nine separate hospitals, (2) centralising and improving thrombolysis treatment to four, and (3) two hospitals. Outcomes were annual mean and incremental costs per patient up to the treatment with thrombolysis, incremental cost-effectiveness ratio (iCER) per 1% increase in thrombolysis rate, and the proportion treated with thrombolysis.

**Results:**

Compared to current decentralised care, improving stroke care at individual community hospitals led to mean annual costs per patient of $US 1,834 (95% CI, 1,823–1,843) whereas centralising to four and two hospitals led to $US 1,462 (95% CI, 1,451–1,473) and $US 1,317 (95% CI, 1,306–1,328), respectively (*P* < 0.001). The iCER of improving community hospitals was $US 113 (95% CI, 91–150) and $US 71 (95% CI, 59–94), $US 56 (95% CI, 44–74) when centralising to four and two hospitals, respectively. Thrombolysis rates decreased from 22.4 to 21.8% and 21.2% (*P* = 0.120 and *P* = 0.001) in case of increasing centralisation.

**Conclusions:**

Centralising thrombolysis substantially lowers mean annual costs per patient compared to raising stroke care at community hospitals simultaneously. Small, but negative effects on thrombolysis rates may be expected.

**Electronic supplementary material:**

The online version of this article (doi:10.1186/s12874-016-0275-3) contains supplementary material, which is available to authorized users.

## Background

Treatment with thrombolysis or tissue plasminogen activator (tPA) in stroke centres as part of a centralised organisational model is associated with better patient outcomes and higher thrombolysis rates compared to community hospitals in a decentralised model [1]. In addition to better patient outcomes, centralisation of thrombolysis may lead to substantial cost-savings [2, 3]. Compared to stroke care at community hospitals, admission to a stroke centre was associated with an incremental cost-effectiveness ratio of US$ 24,000 per quality-adjusted life year gained [4]. For every 5% absolute increase in thrombolysis rates, an additional 30,000 patients annually may be treated within a US region containing 109 designated stroke centres. Within the Netherlands, a Breakthrough Series-based implementation program increased thrombolysis use and saved short- and long-term healthcare costs due to lower hospital admission and residential costs, and increasing stroke care efficiency [5]. The short-term costs and resource implications associated with advancing community hospital stroke care to the standards of a stroke centre however remain unclear, hampering broad implementation of thrombolysis.

Because distances to hospitals offering thrombolysis in the Netherlands are relatively short [6], decentralised stroke systems may be improved in two ways: (1) raising stroke care to the standards of a stroke centre in all individual community hospitals simultaneously, and (2) centralising and simultaneously improving thrombolysis treatment thereby reducing the number of community hospitals offering thrombolysis. Prior to large-scale implementation, the clinical, financial and personnel implications of both scenarios should be assessed. In addition, because the efficacy of thrombolysis is strongly time-dependent (i.e., the sooner the better) [7], we considered how time to treatment and travel time to the hospital would influence patient outcomes.

Using a simulation model, the aims of this study were: (1) to estimate the short-term costs, up to treatment with tPA, and the incremental cost-effectiveness ratio associated with raising stroke care at all community hospitals simultaneously compared to centralisation of thrombolysis treatment and (2) to estimate the effects of centralisation on the average days of extra healthy life, proportion of patients treated with thrombolysis, total process time, and travel time.

## Methods

The present study was based on a previously published 6-month prospective study on a centralised (*n* = 280 of which 124 thrombolysis candidates) and decentralised (*n* = 801 of which 227 thrombolysis candidates) organisational model of acute stroke care within a Dutch region [1]. A summary on patient recruitment, baseline patient characteristics, population densities, and access to healthcare services is provided elsewhere [1]. Variables were obtained from patients admitted to hospitals during a 6 month prospective study from February to July 2010. Data collection focused on time delays along both the pre- and intrahospital acute stroke pathway, and on diagnostic accuracy such as choice for first responder and ambulance transportation.

### Organisational models of acute stroke care

In the North of the Netherlands, a centralised and decentralised organisational model co-exist. The decentralised model comprises nine community hospitals in which tPA treatment is provided to patients in their catchment area. Community hospitals included in this study were medium to large hospitals with 100 to 400 stroke patients admitted per year. University Medical Centre Groningen (UMCG) acting as stroke centre served as an example of centralised stroke care. Acute stroke pathway set-up was identical for both organisational models. The stroke centre has 24-h, 7-day acute stroke care coverage, including immediate access to neuroimaging (CT, Computed Tomography scan) and examination at the Emergency Department (ED). Within all hospitals offering thrombolysis, treatment was given within a stroke unit according to identical protocols for tPA treatment (adjusted ECASS III) [8]. Distances and access to healthcare services such as General Practitioners (GP) offices and Emergency Medical Services (EMS) are typically short. EMS protocols were available for GP offices, ambulance dispatch centres and ambulance personnel [9, 10]. The population density and distribution were roughly similar for the centralised- and decentralised organisational models, i.e., 250 and 190 inhabitants per km^2^, respectively. Within the region a well-developed and thin branched road network exists with minimal traffic congestion.

### Simulation model

**Fig. 1:**
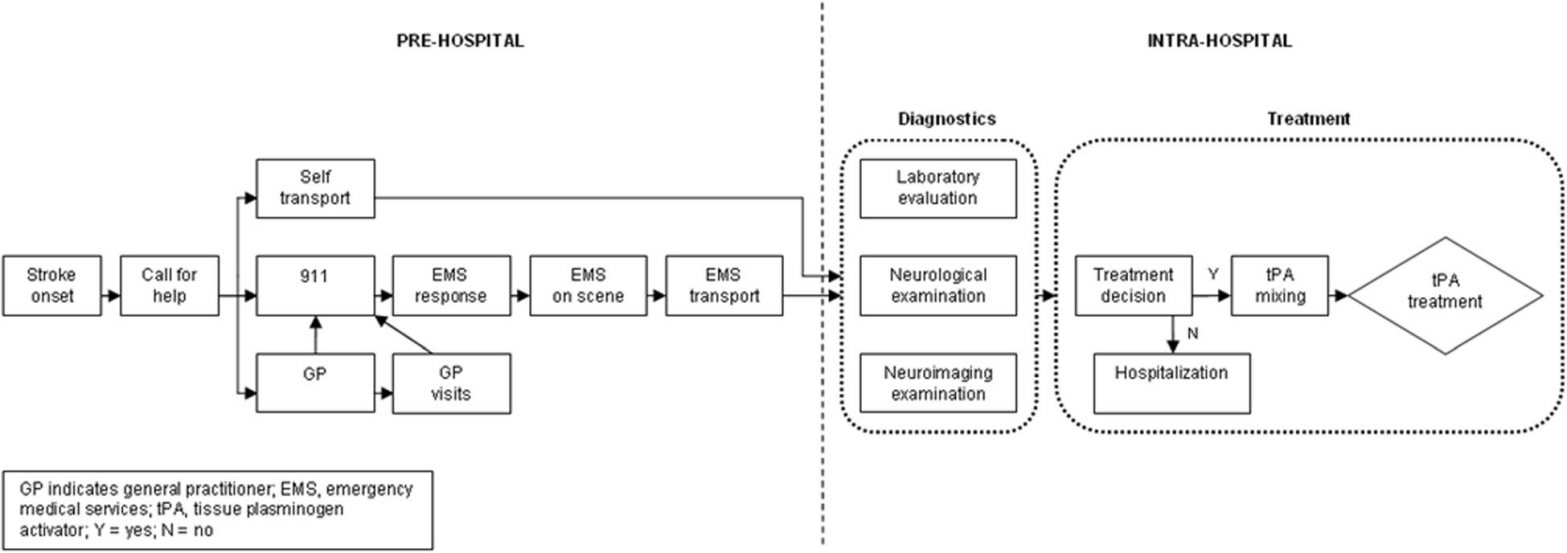
Acute stroke pathway. All key activities that were modeled are depicted

**Fig. 2:**
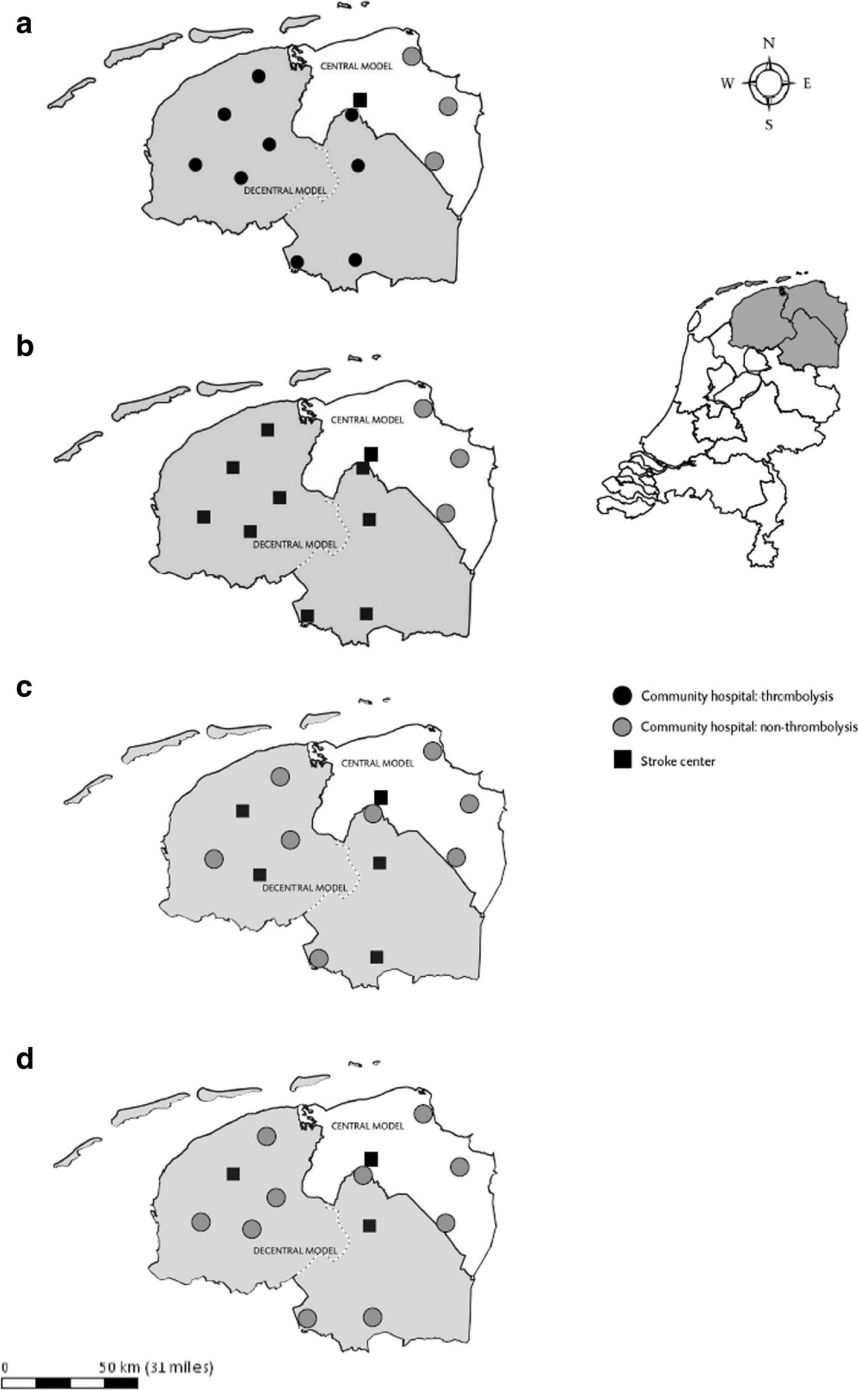
Acute stroke care set-up scenario’s. Current organisational models for acute stroke care in the Northern part of the Netherlands. Within the centralised model thrombolysis is only given in the University Medical Centre Groningen acting as a stroke centre. Arrangements were made with surrounding community hospitals (grey circles) to transport suspected acute stroke patients directly to the stroke centre. The decentralised model consists of nine community hospital all providing thrombolysis within their catchment area (**a**). Improving acute stroke care at all community hospital in the decentralised model to the level of a stroke centre (**b**). Centralisation of the decentral model from nine to four community hospitals (**c**). Centralisation of the decentral model from nine to two hospitals (**d**)

A discrete-event simulation model was designed using Plant Simulation software [11] to replicate current practice of community hospitals based on parameter estimations obtained in the prospective study [1]. A previously developed simulation model was used and extended to represent input data from the decentralised model [12]. Acute stroke pathway set-up is depicted in Fig. 1. Time delays and diagnostic processes were modeled along the pre-hospital and in-hospital stroke pathway using statistical distributions as observed (Table 1). Statistical distributions were determined (fitted) using ExpertFit [13, 14]. Costs associated with resource use were accounted for in the model. Compared to current decentralised care, three scenarios for improving decentralised stroke care were considered from the perspective of the policy maker: (1) simultaneously improving stroke care at all community hospitals, (2) centralisation and improvement of acute stroke care in four, and (3) in two community hospitals, thereby reducing the number of community hospitals offering thrombolysis (Fig. 2). The community hospitals hypothetically acting as stroke centres were chosen based on their geographical location within a region. The UMCG also participated in these scenarios as stroke centre. For the last two scenarios the effect of centralisation on travel time to the treating hospital was assessed. In all scenarios the level of stroke care at hospitals was assumed to rise to the performance of the centralised model; i.e., 22% of all ischaemic stroke patients would be administered thrombolysis [1]. Centralisation only affects those patients for whom delay from stroke onset to the moment of transportation is within the window of opportunity for tPA treatment, i.e., within 4.5 h. All patients facing a delay of more than 4.5 h were sent to the nearest community hospital.

**Table 1:** Distributions specifying activity durations and diagnostic characteristics for the current decentralised model

Activity duration (minutes)				
Model parameter	Distribution: type	Parameters		
Time from stroke onset to call for help	Continuous empirical			
Route 1		Left bound	Right bound	Frequency
		0	5	39
		5	10	21
		10	15	15
		15	30	35
		30	45	33
		45	60	14
		60	120	40
		120	180	16
		180	240	10
		240	480	9
		480	2880	230
Route 2		0	5	1
		5	10	1
		10	15	1
		480	2880	15
Route 3		0	60	9
		60	120	17
		120	180	3
		180	240	4
		240	480	2
		480	2880	286
Delay first responder				
911 call	Uniform	Min (1.00), Max (2.00)		
GP consult by telephone	Uniform	Min (2.00), Max (5.00)		
GP consult by visit	Triangle	Mode (40.00), Min (10.00), Max (30.00)		
EMS				
Response time				
A1	Continuous empirical	Left bound	Right bound	Frequency
		0	5	53
		5	10	145
		10	15	85
		15	20	23
		20	25	2
		25	30	1
		30	35	0
		35	40	2
A2	Gamma	Alpha (3.36), Beta (4.22)		
B	Beta	Alpha 1 (0.69), Alpha 2 (0.53), a (8.92), b (59.33)		
Time spent on scene				
A1	Continuous empirical	Left bound	Right bound	Frequency
		0	5	3
		5	10	45
		10	15	106
		15	20	87
		20	25	39
		25	30	13
		30	35	8
		35	40	3
		40	45	4
		45	60	2
A2	Lognormal	Mean (15.24), St. dev. (7.67)		
B	Lognormal	Mean (15.12), St. dev. (8.14)		
Transport time				
A1	Beta	Alpha1 (1.69), Alpha2 (4.16), a (39.66), b (0.62)		
A2	Gamma	Alpha (5.53), Beta (2.51)		
B	Beta	Alpha 1 (1.07), Alpha 2 (1.41), a (0.02), b (31.61)		
Time to neurological consultation	Continuous empirical	Left bound	Right bound	Frequency
		0	0	79
		0	1	5
		1	2	13
		2	5	39
		5	10	30
		10	15	19
		15	30	12
		30	72	19
Time to neuroimaging (CT) examination	Continuous empirical	Left bound	Right bound	Frequency
		0	5	9
		5	10	23
		10	15	36
		15	20	29
		20	25	32
		25	30	29
		30	35	21
		35	40	11
		40	45	11
		45	50	3
		50	55	2
		55	60	7
		85	90	3
		90	135	2
Time to laboratory examination	Continuous empirical	Left bound	Right bound	Frequency
		0	0	61
		0	10	5
		10	15	8
		15	20	14
		20	25	18
		25	30	16
		30	35	14
		35	40	20
		40	45	18
		45	50	12
		50	80	16
Treatment decision	Triangle	Mode (10), Min (5), Max (20)		
tPA mixing	Constant	5		
Diagnostics				
Choice of route	Discrete empirical	Value		Frequency
1. EMS transport		1		462
2. In-hospital		2		18
3. Self-transport		3		321
Choice first responder	Discrete empirical	Value		Frequency
1. 911 call		1		184
2. GP consult by phone		2		56
3. GP consult by visit		3		126
EMS transport, level of urgency	Discrete empirical	Value		Frequency
911 call				
1. A1		1		92
2. A2		2		7
3. B		3		1
GP consult by telephone				
1. A1		1		60
2. A2		2		39
3. B		3		1
GP consult by visit				
1. A1		1		47
2. A2		2		42
3. B		3		11

Route 1, 2, and 3 indicate patients transported by emergency medical services, those suffering a stroke in the hospital, and patients arriving by self transport, respectively; *GP* General practitioner, *EMS* Emergency medical services; A1, A2, B indicate normative values for ambulance arrival within 15, 30, and > 30 min from the 911 call until arrival at the location of the patients, respectively; CT, computed tomography; tPA, tissue plasminogen activator. Neurological examination, neuroimaging, and laboratory examination are considered parallel activities

Improvements associated with centralisation of thrombolysis treatment were implemented in the simulation model. Pre-hospital factors modeled included: lapse between stroke onset and call for help, GP consultation, EMS use, and high priority ambulance transportation (i.e., arrival within 15 min after alert). Data collected in the prospective study included the travel time from the exact geographical location of the patient to the hospital providing thrombolysis for all patients transported by EMS. In-hospital factors included time from hospital arrival to neurological examination, neuroimaging (CT scan), laboratory examination, and treatment with thrombolysis. In the model 10,000 patients progressed along the stroke pathway. Additional file 1: Tables S1 and S2 describe model parameters for the improved decentralised model (9 hospitals), and effects of centralisation on choice of distributions representing patient transport from the incident scene to a designated hospital (4, 2 hospitals). Further details on simulation methodology, model validation, and model data are provided in Additional file 1: Methods.

### Cost calculation

The costs associated with resource use along the stroke pathway for fixed and variable costs are presented in Table 2. Short-term costs up to treatment with thrombolysis were considered, because these were available. Data was collected on resource use at the level of all individual patients in both the pre-hospital and intra-hospital phase of the stroke pathway. Unit costs were entered into the simulation model and contributed to overall resource use.

**Table 2:** Unit costs for resource utilization

Resource	Unit costs (USD)	Source
Variable costs		
General practitioner		(1)
Telephonic consultation	$19.04	
Visit by general practitioner	$56.00	
Emergency medical services transport		(2)
Emergency transport	$882.00	
Dispatch	$71.00	
Per driven kilometer	$5.00	
Medical personnel ER visit		(1)
Medical specialist (15 min)	$44.38	
Resident (1 h)	$36.48	
Nurse (1 h)	$35.04	
Outpatient clinic visit	$89.60	(1)
Computed tomography scan	$144.48	(3)
Central laboratory (per test)	$27.10	(4)
Alteplase	$532.46	(5)
Fixed costs		
Public education campaigns (range)	$3,750 ($2,500–$5,000)	(6)
Staff education (range)	$7,500 ($5,000–$10,000)	(6)
Computed tomography scan		(7)
Purchase computed tomography scan	$1,310,000	

USD indicates United States dollar; ER, emergency room. (1) Health Care Insurance Board (CVZ) [31]; (2) Data from regional ambulance services Groningen; (3) Dirks et al., 2012 [4]; (4) Claes et al., 2006 [32]; (5) www.medicijnkosten.nl; (6) Alberts et al., 2011 [33]; (7) https://www.medischcontact.nl/nieuws/laatste-nieuws/artikel/weldoener-koopt-ct-scanner-voor-ziekenhuis.htm

Mean and incremental annual costs per patient were estimated for all scenarios including fixed and variable costs. Fixed costs were considered constant whereas variable costs fluctuated directly with patient volumes. Fixed costs included recurring annual public education campaigns and staff education. The purchase of a new CT scanner located in the ED was considered a one-time investment. Yearly depreciation costs for a new CT scanner were conservatively estimated at 10% of the initial investment [15]. Variable costs estimated included GP consultation either by telephone or visit, EMS utilisation, staff deployment associated with activation of the acute stroke team including a stroke neurologist, resident neurology, stroke nurse and treatment with thrombolysis (alteplase). All patients irrespective of eligibility for thrombolysis underwent neurological examination, CT scanning, and laboratory examination, either in the ED for those arriving within the 4.5 h time window or in the outpatient clinic. No additional staff deployment (emergency physicians, neurologists, radiologists or nurses) was anticipated in case of centralisation of thrombolysis, based on expert judgment. Costs per ambulance ride include tariffs for emergency transport, EMS dispatch, and costs per driven kilometer. Costs for deployment of medical personnel per hour included a 39% bonus for social gratuity, holiday pay, and other. To allow estimating annual costs and patient throughput the 6-month study period was extrapolated to 1 year assuming similar resource utilisation serving 1602 patients in the decentral model. The costs in euro’s were adjusted to the current euro-dollar exchange index of 1.12 $US per 1 Euro [16]. Mean resource consumption per patient is presented in Additional file 1: Table S3.

### Travel time and distance

Travel time and distance in case of centralisation of thrombolysis treatment was assessed by hypothetically transporting patients from the emergency site to the nearest hospital offering thrombolysis. Only those patients transported by EMS were included in this analysis, because of availability of data on exact geographical locations for this group. In the scenario of centralisation of thrombolysis, travel time and distance were calculated with the use of a Web based route planner (http://route.anwb.nl/routeplanner) as the product of estimated distances and projected travel speeds, consistent with strategies used in previous studies [17, 18]. The values obtained by the Web-based route planner were corrected to represent real-world data, as the route planner does not account for faster driving speed achieved by ambulance transportation.

### Outcomes measures

The primary end-points were mean and incremental costs per patient associated with all scenarios. Incremental cost-effectiveness ratios (iCERs) concerning the changes in effects (chance of being thrombolysed) and costs associated with improvement and centralisation scenarios vs. the current decentralised model were calculated. Secondary end-points, using the simulation model, included Onset to Treatment Time (OTT), estimations of extra healthy life days calculated using OTT estimates obtained from the simulation model and recently published results on the effects of reducing OTT on extra healthy life [19], the effect of additional travel time in case of centralisation on thrombolysis rates, those thrombolysed within 1.5 h, and the OTT. The OTT is of interest because the efficacy of thrombolysis is time-dependent, i.e., sooner translates into better functional outcome and long-term health benefits (extra healthy life years), in which each minute reduction in OTT results in an average 1.8 days of extra healthy life [20–22]. In addition again based on the OTT we estimated the functional outcomes in terms of the modified Rankin scale. The modified Rankin Scale score is a commonly used scale to measure disability and independence in stroke victims [23]. The scale consists of six grades, from 0 to 5, with 0 corresponding to no symptoms and 5 corresponding to severe disability. Furthermore, we performed a sensitivity analysis to determine how a relative 25% increase or decrease in travel time would influence results in terms of thrombolysis rates, those thrombolysed within 1.5 h, and the OTT.

### Analysis

Costs were assessed separately and presented as means with their corresponding 95% CIs for all scenarios. iCER confidence intervals were estimated using a non-parametric bootstrap method [24], thereby building on simulation output data available for each of the scenarios. Travel times and distances were presented as medians with their corresponding 95% CIs. Mann-Whitney U and Fisher’s exact tests were performed for continuous and categorical variables. SPSS 20.0 for Windows software package (Chicago, IL) was used. A *p*-value < 0.05 was considered statistically significant.

### Informed consent

Informed consent was obtained from all subjects participating in the prospective study [1] and extended for current use. The study was approved by the institutional review board of UMCG.

## Results

### Primary outcomes

Mean annual costs per patient for current decentralised care are $US 922 (95% CI, 911–934). Compared to current decentralised care, improving stroke care at community hospitals separately led to mean annual costs per patient of $US 1,834 (95% CI, 1,823–1,843). Centralising thrombolysis led to a mean annual costs of $US 1,462 (95% CI, 1,451–1,473) when centralising to four, and $US 1,317 (95% CI, 1,306–1,328) when centralising to two hospitals (*P* < 0.001), respectively. The iCER for improving stroke care at all nine community hospitals was $US 113 (95% CI, 91–150) per % increase in thrombolysis rate compared to $US 71 (95% CI, 59–94) and $US 56 (95% CI, 44–72) when centralising to four and two hospitals.

### Secondary outcomes

Table 3 describes the results of the three scenarios performed with the simulation model. Compared to current decentralised care, optimising stroke care at all nine community hospitals resulted in an average 27.0 days of extra of healthy life (95% prediction interval 13.5–40.5), compared to 21.6 days (95% prediction interval 10.8–32.4) and 16.2 days (95% prediction interval 8.1–24.3) when centralising to four and two stroke centres, respectively.

**Table 3:** Results simulation experiments

Scenario	tPA rate (95% CI)	tPA 0–90 min	tPA 90–180 min	tPA 180–270 min	mRS 0-1^a^	OTT minutes (95% CI)	Extra healthy life days (95% prediction interval)^b^
0. Current decentralised stroke care	14.4% (13.7%–15.1%)	14.3%	70.5%	15.2%	14.7%	134 (131–136)	
1. Optimising all 9 Community hospitals	22.4% (21.6%–23.2%)	27.5%	62.0%	10.5%	26.6%	119 (117–127)	27.0 (13.5–40.5)
2. Centralisation (4 stroke centers)	21.8% (21.0%–22.7%)	25.1%	63.2%	11.7%	25.3%	122 (120–124)	21.6 (10.8–32.4)
3. Centralisation (2 stroke centres)	21.2% (20.4%–22.0%)	21.6%	66.6%	11.9%	23.8%	125 (123–127)	16.2 (8.1–24.3)

tPA indicates tissue plasminogen activator; CI, confidence interval; mRS, modified rankin scale; OTT, onset treatment time

^a^Indicates the proportion of patients with excellent functional outcome (mRS 0–1) ascribed with thrombolysis treatment [12]

^b^Indicates the number of additional days in healthy life per minute reduction in OTT [11]

Baseline travel times and distances in case of centralisation are presented as medians with their 95% CIs in Table 4. Overall, centralisation of thrombolysis treatment resulted in travel times of 16.0 (95% CI, 3.0–31.9) and 21.2 min (95% CI, 4.0–37.7) in case of four- and two community hospitals, respectively, compared to 12.0 min (95% CI, 2.0–30.0) in the baseline model (*P* < 0.001). Travel distance increased to 19.7 km (95% CI, 1.1–42.5) and 26.4 km (95% CI, 1.4–54.5), respectively, compared to 11.6 km, 95% CI, 0.9–31.0) in the baseline model (*P* < 0.001).

**Table 4:** Travel times and distances for the baseline case and centralisation

All patients	Optimising all 9 community hospitals	Centralisation (4 stroke centres)	Centralisation (2 stroke centres)
Estimated travel time			
N	446	446	446
Median (95% CI)	12 (2–30)	16 (10–22)†	21 (15–26)†
< 5 min (%)	86 (19)	47 (10)	32 (7)
5–25 min (%)	337 (76)	333 (75)	278 (62)
> 25 min (%)	23 (5)	66 (15)	136 (31)
Estimated travel distance			
Median (95% CI)	12 (1–31)	20 (1–43)†	26 (1–55)†
< 5 km (%)	140 (31)	91 (20)	69 (16)
5–25 km (%)	262 (59)	221 (50)	139 (31)
> 25 km (%)	44 (10)	134 (30)	238 (53)

CI indicates confidence interval

### Sensitivity analysis

In case of centralisation of thrombolysis to four hospitals, a decrease of 25% in travel time increased the proportion of patients treated with thrombolysis with 0.4% (from 21.8 to 22.2%, *P* = 0.544), those thrombolysed within 1.5 h increased from 25.1 to 28.2% (*P* = 0.021), and OTT decreased from 121.9 to 119.1 min (*P* = 0.048). An increase of 25% in travel time decreased the proportion of patients treated with thrombolysis by 0.4% (from 21.8 to 21.4%, *P* = 0.477), those thrombolysed within 1.5 h decreased from 25.1 to 22.4% (*P* = 0.038), and the OTT increased from 121.9 to 124.7 min (*P* = 0.057).

In case of centralisation of thrombolysis to two hospitals, a decrease of 25% in travel time increased the proportion of patients treated with thrombolysis with 0.5% (from 21.2 to 21.7%, *P* = 0.344), those thrombolysed within 1.5 h increased from 21.6 to 25.1% (*P* = 0.001), and OTT decreased from 124.5 to 120.9 min (*P* = 0.001). An increase of 25% in travel time decreased the proportion of patients treated with thrombolysis by 0.6% (from 21.2 to 20.6%, *P* = 0.311), those thrombolysed within 1.5 h decreased from 21.6 to 19.4% (*P* = 0.088), and OTT increased from 124.5 to 127.7 (*P* = 0.027).

## Discussion

This study demonstrated that centralisation of thrombolysis may lead to substantial annual cost-savings per patient compared to a scenario in which stroke care would be improved at all separate community hospitals. Mean costs were lowest when reducing the number of community hospitals offering thrombolysis from nine to two facilities.

The iCER analyses indicated that marginal gains of improving additional community hospitals comes at increasing costs. Furthermore, extended dominance was observed for the less centralised stroke care scenario. Estimating the costs associated with a 1% improvement in tPA treatment rate, and the number needed to treat of 1 in 7 patients on average keeps out nursing home (average annual costs well over €50,000) [25], it becomes clear that the investments required would be quickly regained. We assumed comparable effects of expediting thrombolysis treatment on extra healthy life years, i.e., 1 min reduction in total process time translates into one additional day of healthy life for each patient treated with thrombolysis. This was based on similarities between our population [1] and the one described in the literature [19] in terms of demographics and outcome distributions (modified Rankin Scale scores). When compared to a hypothetical optimised decentral system centralisation of thrombolysis was projected to lead to small, but statistically significant negative effects on the proportion patients treated with thrombolysis, OTT, and fewer additional healthy life years. Whether the better secondary outcomes attained with the optimised decentral system would outweigh the greater costs in terms of long-term health and outcomes remains unclear and is subject of future study. As estimating iCER in terms of cost per year free of stroke symptoms would rely on additional assumptions and thus introducing further uncertainty we refrained from estimating this outcome. Long-term effects were not taken into consideration as the long-term consequences slight increases OTT are difficult to interpret. In any case, this suggests that centralising thrombolysis treatment should be accompanied by initiatives to reduce time delays in other parts of the chain of care; for example by reducing the door-needle-time. Importantly, thrombolysis rates in a centralised system would clearly surpass those of current decentralised care. Because of inequalities in geographic and resource availability between regions and facilities, our analysis provides broadly applicable estimates yet might lack generalisability. However, to account for the potential effects of, among others, population density, regional geography, and traffic congestion, and to generalise the findings presented in this study to other regions, a sensitivity analysis on transport times was performed. The sensitivity analyses demonstrated that moderate changes in travel times did not substantially alter results, suggesting robustness of our findings. In addition, by manipulating input model parameters, i.e., performing scenario and sensitivity analyses, for example travel distances, less densely populated areas could also be represented thus assisting in optimising the chain of care in other settings as well.

The results of this study corroborate the relevance of applying organisational models for the configuration of acute stroke care for a region, instead of local improvements in an individual chain of care. Stroke centre designation will impact on hospital services in several ways. First, available resources may be re-distributed if the hospital needs to purchase expensive equipment, hire more staff, or expand bed capacity. The question whether additional staff is needed to match an increase in patient volumes when thrombolysis treatment is centralised remains speculative, and further evidence is urgently needed. Assuming an annual gain in thrombolysis candidates of 16% as a result of centralisation, staff members may be deployed an additional 256 times annually, from 449 (28% of 1602 stroke patients) to 705 (44% of 1602 patients). In case the appropriate equipment and staff are already available, changes may only be necessary in terms of the time of day staff will be on call, i.e., more hours into a stroke team. This would imply that the capacity of stroke centres does not require expanding despite an increase in patient load. Whether or not this is realistic is subject of further study. Improving coordination of care through stroke centre designation reduces duplication of efforts and redundant diagnostic testing. In particular, less personnel, work hours, and materials are needed. Therefore limited hospital budgets only have to be spent once in a centralised setting instead of raising stroke care at all community hospitals separately. Furthermore, being a stroke centre likely will increase the use of other hospital departments and services, i.e., radiology and laboratory services, which may result in increased revenues [26].

Unavoidably centralisation leads to longer travel times from the emergency site to the hospital offering thrombolysis. Yet, as demonstrated in a previously published study this can be compensated by shorter intra-hospital processes (door-to-needle time) of 35 min compared to 47 min in a decentralised model [1]. In addition, previous research showed that the door-to-needle time may be further shortened to 20 min in optimised settings [27, 28]. We assumed that centralising stroke care may lead to a doubling of emergency rides by EMS; this would not require hiring additional EMS personnel or purchasing additional vehicles. However this too remains a topic for further study.

Our study has limitations. First, as a short-term time horizon, i.e., only the costs up to treatment with thrombolysis were considered, effects of e.g., postponed therapy such as long-term sequelae of ischaemic strokes could not be taken into account. Future studies should try to include long-term data in their analyses, or use historical data from review articles. Secondly, costs associated with thrombolysis such as antithrombotic and lipid lowering medications were not part of the assessment and could therefore not be controlled for. Future studies should try to collect these variables prospectively and include them in the costs analyses. Third, we mostly used tariffs rather than societal costs for our analyses. Also, the effects of improved patient outcomes on the frequency and intensity of informal (family) care should be assessed. An additional concern is the potential for traffic congestion which might influence estimated travel times in our study. However, all patients included in this study had access to 911-systems, and previous research indicated that traffic patterns only minimally affects ambulance travel times [29]. Finally, we did not consider the possibility of misdirecting patients to decentralised hospitals based on incorrect EMS assessments. However, both recent research [30], and our own prospective data on the studied region indicate how suchlike misinterpretations involve only 1–2% of the patient population.

## Conclusions

Centralisation of thrombolysis leads to substantially lower annual costs per patient compared to raising the level of stroke care at community hospitals simultaneously. Small, but negative effects on average days of healthy life, thrombolysis rates, and OTT may be expected compared to optimised decentral care.

## Additional file

Additional file 1: Table S1. Distributions specifying activity durations and diagnostic characteristics for the optimised decentralised model in case of centralisation. **Table S2.** Model parameter distributions for emergency medical services transport to hospital in case of centralisation. **Table S3.** Mean resource consumption per patient for the different scenarios. (DOCX 47 kb)

## Abbreviations

CT: Computed Tomography
ED: Emergency Department
EMS: Emergency Medical Services
GP: General Practitioner
iCER: Incremental Cost Effectiveness Ratio
OTT: Onset Treatment Time
tPA: Tissue plasminogen activator
UMCG: University Medical Center Groningen
