# Supplementary material for: Centralising and optimising decentralised stroke care systems: a simulation study on short-term costs and effects
Source: BMC Med Res Methodol. 2017 Jan 10;17:5. doi: 10.1186/s12874-016-0275-3 (PMC5223548; doi:10.1186/s12874-016-0275-3)
Supplement: Additional file 1: Table S1. — Distributions specifying activity durations and diagnostic characteristics for the optimised decentralised model in case of centralisation. Table S2. Model parameter distributions for emergency medical services transport to hospital in case of centralisation. Table S3. Mean resource consumption per patient for the different scenarios. (DOCX 47 kb) [file 12874_2016_275_MOESM1_ESM.docx]

**ONLINE DATA SUPPLEMENT**

**Centralising and optimising decentralised stroke care systems:**

**A simulation study on short-term costs and effects**

Maarten M.H. Lahr Ph.D.

Durk-Jouke van der Zee Ph.D.

Gert-Jan Luijckx M.D., Ph.D.

Patrick C.A.J. Vroomen M.D., Ph.D.

Erik Buskens, M.D., Ph.D.

Introduction: page 2

Supplemental Methods: page 2

Supplemental Results: page 3

**Introduction**

The main text of the manuscript summarises and discusses the most important findings of the study. This online supplement provides details on simulation modelling methodology and model data.

**Supplemental Methods**

***Discrete event simulation***

The simulation model built conforms to the notion of Discrete Event Simulation (DES). DES entails the modelling of a system as it evolves over time by a representation in which variables change instantaneously at separate, i.e. discrete, points in time. Key activities included model building, validation, and performing experiments. Because the acute stroke pathway incorporates a great deal of variation in activity durations and diagnostics [1], DES is considered an appropriate method for modelling such processes.

***Model validation***

The model has been validated by considering real system performance, i.e. thrombolysis rate in the decentral model was 14.4% vs. 14.1% in the real system (P=0.87), and onset-treatment-time was 127 vs. 134 minutes in the real system (P=0.12). Next, we implemented success factors of the centralised model by adjusting model data. Respective factors were validated in a previous study [2]. Face validity was supported by two stroke neurologists (G.J.L., and P.C.A.J.V.) acting as domain experts. Performance of the adjusted model and the centralised model were similar, i.e. 22.4% versus 22.1%, respectively.

***Distribution fitting***

eTables 1 and 2 specified which parameter changes in case of centralisation. Main steps involved:

- Importing real system data into ExpertFit [3].
- Fitting theoretical distributions by using the method of maximum likelihood [1].
- Seeking further evidence in case of a “no fit”, in an attempt to underpin the choice for a specific theoretical distribution. Evidence considered includes usage of the candidate distribution(s), commonalities between highest ranked distributions, and consultation of domain experts [4]. If such evidence is not found an empirical distribution was chosen.

***Set-up of experiments***

Three scenarios are considered, see main text. Table e1 describes to which degree parameters were changed in case of centralisation. eTable 2 supplies data on alternative distributions for transportation times, as implemented for scenarios 2 and 3. All experiments concern observations on 10,000 hypothetical patients.

***Software***

Plant Simulation was used to model the stroke pathway [5]. Choice of probability distributions and their respective parameters is made using ExpertFit [3].

***Model data***

Traversing each route entails sampling from distributions specifying activity durations. Note how activity durations may be moderated by diagnostic outcomes. Route 1, 2, and 3 indicate patients transported by emergency medical services, those suffering a stroke in the hospital, and patients arriving by self transport, respectively.

**Supplementary Results**

***Mean resource consumption per patient***

Mean resource consumption per patient for the different scenarios is presented in Table e3.

***Treatment decision***

The efficacy of thrombolysis in acute ischemic stroke is greater the earlier it is administered, and the clinical benefit declines progressively over time [6]. For the simulation model the likelihood of treatment is approximated by a linear function, see eFigure 1. We used a linear regression model (Y-axis intercept 82.1; slope -0.25) to approximate the chance of tPA treatment set against the overall time delay for all patients arriving < 4.5 hours from the onset of stroke symptoms (i.e. eligible for tPA treatment).

**Table e1.** Distributions specifying activity durations and diagnostic characteristics for the optimised decentralised model in case of centralisation.

| **Activity duration (minutes)** |  |  | | | | | |
| --- | --- | --- | --- | --- | --- | --- | --- |
| Model parameter | **Distribution: type** | **Parameters** | | | | | |
| Time from stroke onset to call for help  Route 1  Route 2  Route 3 | Continuous empirical | Left bound  0  5  10  15  30  45  60  120  180  240  480  0  120  240  480 | | Right bound  5  10  15  30  45  60  120  180  240  480  2880  5  180  480  2880 | | | Frequency  34  4  8  13  15  13  19  13  9  12  73  6  1  1  58 |
| Delay first responder  911 call  GP consult by telephone  GP consult by visit | Uniform  Uniform  Triangle | Min (1.00), Max (2.00)  Min (2.00), Max (5.00)  Mode (40.00), Min (10.00), Max (30.00) | | | | | |
| Emergency Medical Services |  |  | | | | | |
| Response time  A1 | Gamma | Alpha (1.36), Beta (6.29) | | | | | |
| A2 | Lognormal | Mean (14.21), Standard deviation (6.51) | | | | | |
| B | Beta | Alpha 1 (1.70), Alpha 2 (3.54) , a (0.81), b (110.47) | | | | | |
| Time spent on scene  A1 | Gamma | Alpha (1.36), Beta (6.29) | | | | | |
| A2 | Lognormal | Mean (18.11), Standard deviation (8.39) | | | | | |
| B | Lognormal | Mean (14.25), Standard deviation (8.60) | | | | | |
| Transport time  A1  A2  B | Weibull  Weibull  Beta | Alpha (1.93), Beta (19.15)  Alpha (1.43), Beta (16.01)  Alpha 1 (1.32), Alpha 2 (2.56) | | | | | |
|  |  |  |  | | |  | |
| Time to neurological consultation | Continuous empirical | Left bound  0  0  1  2  5 | Right bound  0  1  2  5  24 | | | Frequency  93  4  7  6  12 | |
| Time to neuroimaging (CT) examination | Continuous empirical | Left bound  2  6  11  16  21  31 | Right bound  5  10  15  20  30  56 | | | Frequency  28  54  13  10  8  8 | |
| Time to laboratory examination | Erlang | Mean (32.29), Standard deviation (9.26), Location (2.83) | | | | | |
| Treatment decision | Triangle | Mode (10), Min (5), Max (20) | | | | | |
| tPA mixing | Constant | 5 | | | | | |
|  |  |  | | | | | |
| **Diagnostics** |  |  | | | | | |
| Choice of route  1. EMS transport  2. In-hospital  3. Self-transport | Discrete empirical | Value  1  2  3 | | | Frequency  213  60  7 | | |
| Choice first responder  1. 911 call  2. GP consult by phone  3. GP consult by visit | Discrete empirical | Value  1  2  3 | | | Frequency  30  19  27 | | |
| EMS transport, level of urgency  911 call  1. A1  2. A2  3. B  GP consult by telephone  1. A1  2. A2  3. B  GP consult by visit  1. A1  2. A2  3. B | Discrete empirical | Value  1  2  3  1  2  3  1  2  3 | | | Frequency  95  3  2  88  10  2  60  33  7 | | |

Route 1, 2, and 3 indicate patients transported by emergency medical services, those suffering a stroke in the hospital, and patients arriving by self transport, respectively; GP, general practitioner; A1, A2, B indicate normative values for ambulance arrival within 15, 30, and > 30 minutes from the 911 call until arrival at the location of the patients, respectively; CT, computed tomography; tPA, tissue plasminogen activator; EMS, emergency medical services. Neurological examination, neuroimaging, and laboratory examination are considered parallel activities.

**Table e2.** Model parameter distributions for emergency medical services transport to hospital in case of centralisation.

|  | **Distribution: type** | **Parameters** | | |
| --- | --- | --- | --- | --- |
| **Centralisation (4 hospitals)** |  |  | | |
| Model parameter |  |  | | |
| A1 | Beta | Alpha 1 (1.92), Alpha 2 (2.67) | | |
| A2 | Weibull | Alpha (1.93), Beta (19.81) | | |
| B | Beta | Alpha (1.28), Beta (0.79) | | |
|  |  |  | | |
| **Centralisation (2 hospitals)** |  |  | | |
| Model parameter |  |  | | |
| A1 | Continuous empirical | Left bound  0  5  10  15  20  25  30  35  40 | Right bound  5  10  15  20  25  30  35  40  45 | Frequency  17  34  35  63  97  41  29  6  2 |
| A2 | Weibull | Alpha (2.19), Beta (26.05) | | |
| B | Beta | Alpha (1.98), Beta (1.09) | | |

A1, A2, B indicate normative values for ambulance arrival within 15, 30, and > 30 minutes from the 911 call until arrival at the location of the patients, respectively.

**Table e3.** Mean resource consumption per patient for the different scenarios.

|  | **Current decentralised  care** | **Optimising all 9  community hospitals** | **Centralisation  (4 stroke centres)** | **Centralisation  (2 stroke centres)** |
| --- | --- | --- | --- | --- |
| Variable costs |  |  |  |  |
| General practitioner |  |  |  |  |
| Telephone consultation, (%) | 0.21 | 0.27 | 0.27 | 0.27 |
| Home visit, (%) | 0.48 | 0.40 | 0.40 | 0.40 |
|  |  |  |  |  |
| Emergency medical services |  |  |  |  |
| Emergency transport and dispatch, (%) | 0.58 | 0.77 | 0.77 | 0.77 |
|  |  |  |  |  |
| Medical personnel ER visit, (%) | 0.31 | 0.54 | 0.55 | 0.55 |
| Outpatient clinic visit, (%) | 0.69 | 0.46 | 0.45 | 0.45 |
| Computed tomography scan, (%) | 1.0 | 1.0 | 1.0 | 1.0 |
| Central laboratory test, (%) | 1.0 | 1.0 | 1.0 | 1.0 |
| Alteplase (activated tPA), (%) | 0.14 | 0.22 | 0.22 | 0.21 |

| ER indicates emergency room; tPA, tissue plasminogen activator. |  |  |
| --- | --- | --- |

**eFigure 1.** Treatment decision: a patient’s chance of being treated given the overall time delay.


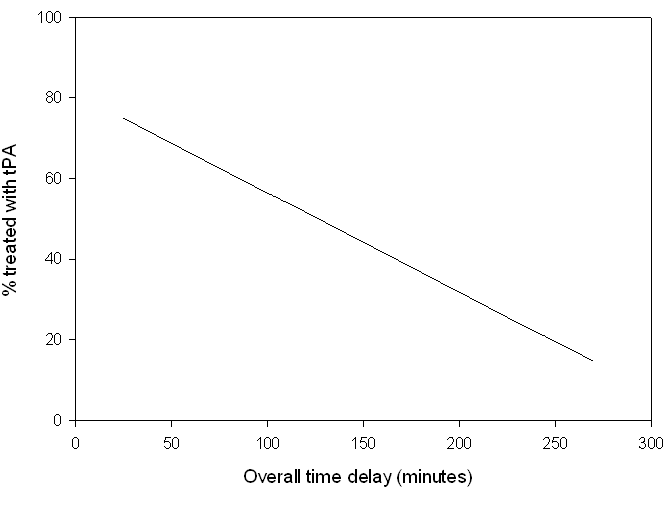


**Supplemental References**

[1] Law A.M., Kelton W.D. 2007. Simulation modelling and analysis. 4th ed. : McGraw-Hill.

[2] Lahr M.M., van der Zee D.J., Vroomen P.C., Luijckx G.J., Buskens E. 2013. [Thrombolysis in Acute Ischemic Stroke: A Simulation Study to Improve Pre- and in-Hospital Delays in Community Hospitals.](http://www.ncbi.nlm.nih.gov/pubmed/24260151) PLoS One. 8(11):e79049.

[3] Law A.M. 2011. ExpertFit Version 8 User´s Guide. Tuscon, Arizona: Averill M. Law & Associates.

[4] Stahl J.E., Furie K.L., Gleason S., Gazelle G.S. 2003. Stroke: Effect of implementing an evaluation and treatment protocol compliant with NINDS recommendations. Radiology;228(3):659-668.

[5] Plant Simulation. Siemens PLM 2012. [accessed March 2, 2016]. Available at: <http://www.plm.automation.siemens.com/en_us/products/tecnomatix/plant_design/plant_simulation.shtml>.

[6] Lees K.R., Bluhmki E., von Kummer R., Brott T.G., Toni D., Grotta J.C., Albers G.W., Kaste M., Marler J.R., Hamilton S.A., Tilley B.C., Davis S.M., Donnan G.A., Hacke W.; ECASS, ATLANTIS, NINDS and EPITHET rt-PA Study Group, Allen K., Mau J., Meier D., del Zoppo G., De Silva D.A., Butcher K.S., Parsons M.W., Barber P.A., Levi C., Bladin C., Byrnes G. 2010. Time to treatment with intravenous alteplase and outcome in stroke: an updated pooled analysis of ECASS, ATLANTIS, NINDS, and EPITHET trials. Lancet;375(9227):1695-1703.
